# Zinc phosphide poisoning with unusual radiologic findings

**DOI:** 10.1002/ccr3.807

**Published:** 2017-02-01

**Authors:** Sayyedmojtaba Nekooghadam, Hamidreza Haghighatkhah, Fateme Vaezi, Morteza Sanei Taheri, Yashar Moharamzad

**Affiliations:** ^1^ Department of Internal MedicineShohada HospitalShahid Beheshti University of Medical SciencesTehranIran; ^2^ Department of RadiologyShohada HospitalShahid Beheshti University of Medical SciencesTehranIran; ^3^ School of MedicineKermanshah University of Medical SciencesKermanshahIran

**Keywords:** Computed tomography angiography, poisoning, rodenticide, shock, zinc phosphide

## Abstract

Accidental/suicidal ingestion of metal phosphides (e.g., zinc phosphide found in rodenticides) should be suspected in patients with sudden‐onset abdominal pain, refractory hypotension, and metabolic acidosis. CT angiography may show radiopaque substance in the stomach and early enhancement of the inferior vena cava and contrast in right side of the heart.

## Introduction

Rodenticides comprise heterogeneous agents with distinctly different toxicity profiles. Metal phosphides such as aluminum phosphide and zinc phosphide (Zn_3_P_2_) are among the most potent rodenticides. In both humans and rodents, gastric acid reacts with phosphide to generate toxic phosphine gas, a highly lethal compound 1, 2.

There are concerning anecdotal reports that ingestion of such agents to commit suicide has observed a growing trend during recent years in Iran. Among scarce reports on this topic available in the literature, only a single particular study reported 102 patients presented with this kind of poisoning to a single referral poisoning center in Tehran, Iran, during a 3.5‐year retrospective study period 3. However, this presentation is not limited to Iran. As these rodenticides are cheap, they are extensively used, especially in developing countries, and several reports have described mortalities as a result of accidental or suicidal ingestion of such compounds, especially in the 1990s in India. Poisoning with metal phosphides has been described as the most prevalent cause of poisoning in rural areas of Northern India 4. Besides, this poisoning has been reported from other countries such as South Africa 5, France 6, Mexico 7, and many other areas.

Many characteristic clinical and laboratory properties of metal phosphide poisoning have been described in toxicology and forensic medicine journals. But, to date, no clinico‐radiologic correlation of such patients has been published.

In previous reports of zinc phosphide poisoning, some important radiographic findings, such as radiopaque nature of the substance, and imaging findings pointing to pulmonary and abdominal complications of intoxication have been described 3, 8.

Here, we will describe some new radiological features in a patient with zinc phosphide poisoning. The constellation of clinic‐radiologic findings described here has not been reported previously in the literature. In our opinion, knowledge of these new findings would help the emergency physicians or toxicologists/radiologists for earlier diagnosis of this usually lethal intoxication and more immediate planning for accurate therapeutic interventions.

## Case Report

A 24‐year‐old man was brought to the emergency department (ED) of our hospital because of suddenly started abdominal pain, altered mental status, and agitation for the past 2 h. He complained of a severe persistent abdominal pain predominantly in the epigastric area. He was so agitated that apart from abdominal pain, no other history could be obtained. His colleague stated that he last noticed that the patient was “completely well” and was walking to the office about 3 h earlier. Then, he added that he was called by the patient's co‐workers as they found the patient agitated while complaining of severe abdominal pain, nausea, stool incontinence, and diarrhea.

Past medical history of the patient was completely unrevealing, and no chronic medicine use or chronic medical condition was reported by his colleague. Social history was also unremarkable, except for occasional cigarette smoking. No illicit drug use was reported.

Initial physical examination showed a young thin man who was in severe distress. His heart rate was 110 beats per min, blood pressure (BP) was was 90/60 mmHg, and respiratory rate was 24/min. Oxygen saturation in ambient air was 89%. Auscultation of the heart revealed a moderate tachycardia with muffled heart sounds. Abdominal examination showed hyperactive bowel sounds and tenderness over the epigastrium with mild distension. No evidence of previous surgical scar was found.

Ultrasonography, according to the RUSH protocol, revealed mild pericardial effusion as well as moderate pleural and peritoneal effusions. ECG, performed about 10 min later, showed ST segment elevations in the leads II, III, and aVF. A presumptive diagnosis of internal hemorrhagic shock was made based on the abovementioned findings.

**Figure 1:**
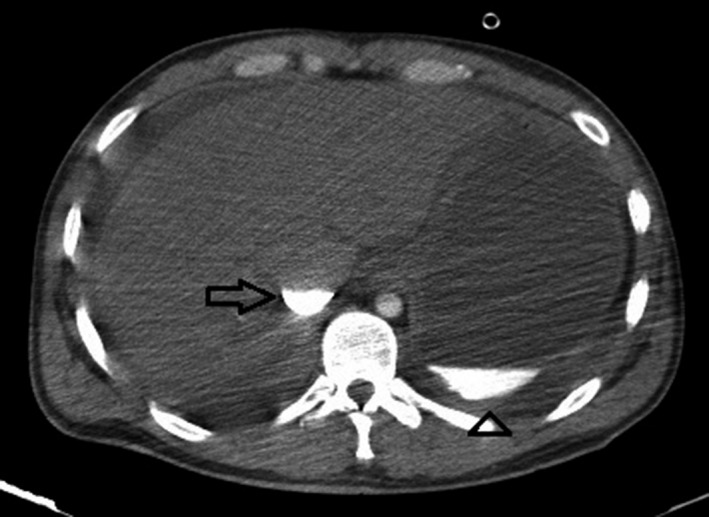
Contrast‐enhanced MDCT angiography shows radiopaque substance in the stomach despite no use of oral contrast (arrowhead). Early enhancement of the IVC (inferior vena cava) during arterial phase of the CT study with contrast‐fluid level formation (arrow) is evident.

**Figure 2:**
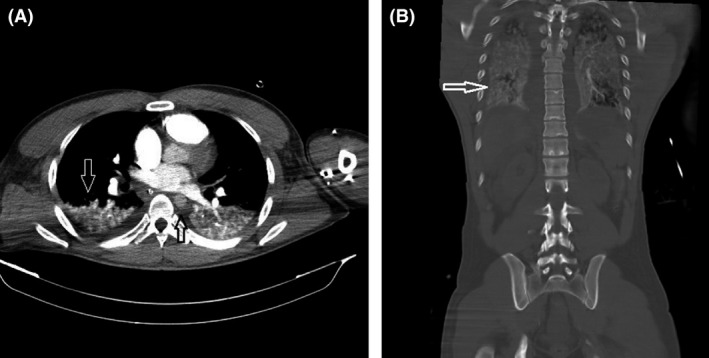
Pulmonary congestion (white arrows in A and B) and small caliber aorta (black arrow in A) are seen. Bilateral pleural effusions and contrast media filling the right side of the heart without evidence of contrast in the left side during arterial phase of the CT study are evident.

The patient underwent emergency contrast‐enhanced CT angiography in a try to find any vascular lesion, especially dissection of the aorta. It revealed no evidence of aortic dissection but unexpectedly showed the presence of a radiopaque substance in the stomach, despite no administration of oral contrast (Fig. 1). Plus, CT angiography revealed RV (right ventricular) failure as indicated by the contrast media filling the right side of the heart without evidence of contrast in the left side of the heart, small caliber aorta without contrast enhancement, and dilatation of the pulmonary veins, IVC (inferior vena cava), and hepatic veins with early enhancement due to contrast reflux. A contrast‐fluid level during the arterial‐dominant phase of the CT study was seen in the IVC; Figure 2.

**Figure 3:**
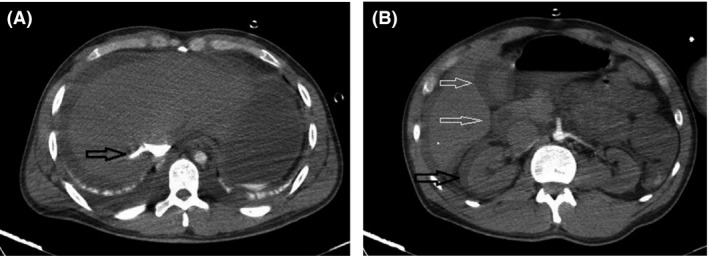
(A) Contrast reflux in the inferior vena cava and hepatic vein is seen (arrow in A). Ascites and bilateral pleural effusion are present. (B) Gall bladder wall and intestinal submucosal edema (white arrows). A large subcapsular fluid collection in the right kidney (black arrow) with decreased attenuation of nephrogram in the cortical phase in both kidneys as a result of decreased renal blood flow is present.

There was increased pulmonary and systemic venous pressure resulting in bilateral pleural effusions, ascites, and submucosal edema in the intestines and gallbladder wall. Attenuation of the nephrogram in cortical phase had been decreased in both kidneys as a result of decreased renal blood flow. A large subcapsular fluid collection was seen in the right kidney (Fig. 3). Paracentesis of abdominal ascites showed a clear yellowish fluid without any evidence of intra‐abdominal hemorrhage.

At this stage, clinical and radiologic findings were in favor of possible oral intoxication with a radiopaque poisonous substance which resulted in RV functional impairment and multiorgan failure. With this presumptive diagnosis in mind, further detailed questioning from his co‐workers revealed that the patient had ingested a suspicious bottle of “unknown” fruit juice shortly before the initiation of his symptoms.

Despite aggressive resuscitation efforts made, the patient developed severe metabolic acidosis 2 h after admission which was refractory to bicarbonate therapy. Meanwhile, his BP started to decline progressively despite receiving intravenous fluids and vasopressor therapy. He was intubated, and mechanical ventilation was initiated. Unfortunately, the patient did not respond to the resuscitative efforts and died about 4 h after admission. Autopsy was performed which confirmed the diagnosis of poisoning with zinc phosphide probably in a suicidal/homicidal scenario.

## Discussion

The most available sources for access to rodenticides, including zinc phosphide or aluminum phosphide, inside Iran are shops where agricultural equipment is sold. Rarely, customers of these substances purchase and use these substances not for agricultural purposes, but ingest them as a way for committing suicide. Combination of zinc with phosphide is known as a rodenticide agent. Zinc, owing to its radiopaque property, is visualized on radiologic examination 8. Committing suicide with phosphide is not unusual 9. Rapid production and absorption of phosphide gas in the stomach is noted after ingestion of zinc phosphide. This gas is a toxic mediator with several lethal effects at cellular level. Impairment of oxidative respiration with decrease in mitochondrial membrane potential can cause several clinical symptoms. Heart and lungs are the most important sites that injury occur 10. Due to impaired myocardial contractility (cardiogenic shock), symptoms progress rapidly and circulatory failure ensues. Acute pulmonary edema and congestive heart failure also can occur. Gastrointestinal complaints such as diarrhea, nausea, and vomiting as well as metabolic acidosis have been reported 1, 11, 12. Signs such as circulatory collapse and some degree of lung damage due to severe hypotension with resistant nonresponsible metabolic acidosis have been reported with zinc phosphide 10.

In a previous case series of two patients, abdominal plain radiography was introduced as a useful diagnostic tool in zinc phosphide poisoning. The authors emphasized that patients with radiopaque abdominal radiographs are more prone to become unstable state even if they are asymptomatic on presentation. Therefore, such patients should be considered as potential candidates for aggressive resuscitative treatments 8.

In our patient, CT angiography was performed to exclude the presumptive diagnosis of dissection of the aorta. However, this radiologic examination revealed phosphide poisoning‐related imaging findings including RV failure with increased pulmonary and systemic venous pressure with early back wash of contrast in the pulmonary and systemic veins. The reason of early enhancement of the IVC was due to pooling and refluxing of the contrast media into the IVC due to circulatory failure. This radiologic sign has been referred to as an imminent sign of cardiogenic shock and death 13. No evidence of contrast was seen in the left side of the heart, and a little amount of contrast media was noted in the aorta. In addition, evidence of multiorgan function impairment, such as infarction in both kidneys due to circulatory collapse, was also seen.

## Conclusion

When facing with a young patient with suddenly started abdominal pain and refractory hypotension, without any previous history of cardiovascular diseases, it is useful to think about intoxication with substances such as zinc phosphide. In such case, abdominal radiography which shows a radiopaque substance as well as detailed and accurate history obtained from the patient or the person who is accompanying the patient would yield valuable information.

## Conflict of Interest

The authors declare that they have no conflict of interest.

## Authorship

SN: provided the conception of the report. HH: reviewed the literature. FV: drafted the article and reviewed the literature. MST: provided the conception and supervised the report, and prepared the images. YM: drafted the article and reviewed the literature.
